# Complete Genome Sequence of the *Gordonia* Bacteriophage BiggityBass

**DOI:** 10.1128/mra.00469-22

**Published:** 2022-08-08

**Authors:** Cyril J. Versoza, Abigail A. Howell, Tanya Aftab, Madison Blanco, Akarshi Brar, Elaine Chaffee, Nicholas Howell, Willow Leach, Jackelyn Lobatos, Michael Luca, Meghna Maddineni, Ruchira Mirji, Corinne Mitra, Maria Strasser, Saige Munig, Zeel Patel, Minerva So, Makena Sy, Sarah Weiss, Christopher D. Herren, Martha Smith Caldas, Susanne P. Pfeifer

**Affiliations:** a School of Life Sciences, Arizona State University, Tempe, Arizona, USA; b Center for Evolution and Medicine, Arizona State University, Tempe, Arizona, USA; c Biodesign Institute, Arizona State University, Tempe, Arizona, USA; d School of Mathematical and Statistical Sciences, Arizona State University, Tempe, Arizona, USA; e School of Molecular Sciences, Arizona State University, Tempe, Arizona, USA; f Division of Biology, Kansas State University, Manhattan, Kansas, USA; g Center for Mechanisms of Evolution, Arizona State University, Tempe, Arizona, USA; Queens College CUNY

## Abstract

Here, we characterized the complete genome of the *Siphoviridae* BiggityBass, a lytic subcluster DR bacteriophage infecting Gordonia terrae CAG3. Its 63.2-kb genome contains 84 protein-coding genes, of which 40 could be assigned a putative function. BiggityBass is related most closely to AnClar and Yago84 with 90.61% and 90.52% nucleotide identity, respectively.

## ANNOUNCEMENT

Bacteriophages are one of the most abundant organisms on our planet (1), and yet, their diversity remains poorly characterized. As part of the Howard Hughes Medical Institute Science Education Alliance–Phage Hunters Advancing Genomics and Evolutionary Science (HHMI SEA-PHAGES) course-based undergraduate research experience, we characterized the complete genome sequence of BiggityBass, an obligatory lytic Gordonia terrae CAG3 bacteriophage.

BiggityBass was obtained by direct plating of a filtered soil sample collected in Manhattan, Kansas, at the top of a hill on the Konza Prairie Nature Trail (39.108056 N, 96.596944 W), purifying a well-isolated plaque, and amplifying the plaque in Gordonia terrae CAG3 (which was propagated in peptone-yeast extract-calcium [PYCa] broth at 30°C for 48 h with aeration in an orbital incubator at 250 rpm), following the procedures outlined in the SEA-PHAGES Discovery Guide (https://seaphagesphagediscoveryguide.helpdocsonline.com/home).

**FIG 1:**
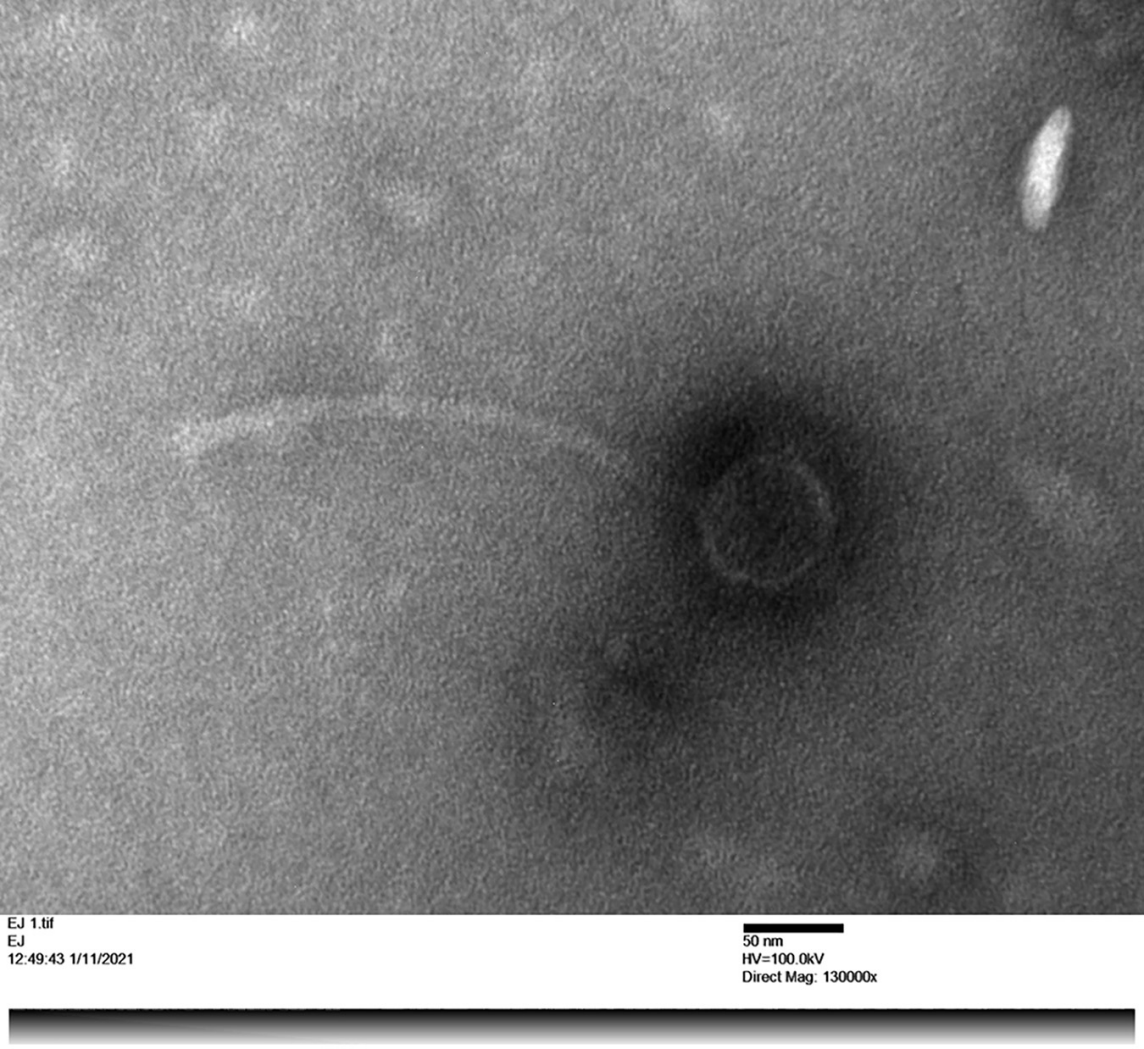
Transmission electron microscopy image showing BiggityBass *Siphoviridae* morphology, characterized by a long, noncontractile tail and an icosahedral capsid that contains the dsDNA.

Copper electron microscopy grids were prepared using uranium acetate staining, which demonstrated that BiggityBass exhibits a *Siphoviridae* morphology, characterized by a long, noncontractile tail and an icosahedral capsid that contains the double stranded DNA (dsDNA) (Fig. 1). Genomic DNA was extracted using the Promega Wizard DNA clean-up kit (washing columns twice with 80% propanol, without RNase or DNase pretreatment), and a library was prepared using the NEBNext Ultra II FS kit and sequenced on an Illumina MiSeq instrument to more than 900-fold coverage. Next, 411,782 high-quality, 150-bp single-end raw sequencing reads were *de novo* assembled using Newbler v.2.9 (2), yielding a complete genome of length 63,202 bp with circularly permuted ends. The genome exhibits a GC content of 69.4%, which is similar to that of its host Gordonia terrae (67.8%). The assembly was checked for completeness, accuracy, and genome termini using Consed v.29.0 (3). All software was executed using default settings.

Following the HHMI SEA-PHAGES Bioinformatics Guide (https://seaphagesbioinformatics.helpdocsonline.com/home), 84 genes were predicted using GLIMMER v.3.0.2 (4) and GeneMark v.2.5 (5) embedded within DNA Master (http://cobamide2.bio.pitt.edu), corresponding to a gene density of 1.31 genes/kb. Out of these 84 genes, 40 could be assigned a putative function using BLASTp v.2.13 (6) and HHPred (7) alignments against GenBank and the Protein Data Base, as well as Phamerator (8) to determine synteny with other cluster DR bacteriophages. Thereby, the position of the minor tail protein (gene 18) is of particular note, potentially hinting at a host range pattern that is different from other cluster DR bacteriophages. In addition, six genes were identified to be membrane proteins using TMHMM v.2.0 (9) and SOSUI v.1.11 (10). ARAGORN v.1.1 (embedded within DNA Master) and v.1.2.38 (11) as well as tRNAscan-SE v.2.0 (12) were used to search for tRNAs and transfer-messenger RNAs (tmRNAs), but none were found.

Multiple sequence alignments using MAFFT v.7 (13) indicated that BiggityBass is related most closely to AnClar (GenBank accession number MN908693) and Yago84 (GenBank accession number MK801725) (14) with a nucleotide sequence similarity of 90.61% and 90.52%, respectively.

### Data availability.

The whole-genome sequencing data are available through NCBI Sequence Read Archive (BioProject accession number PRJNA488469; run number SRR20167194). The annotated genome assembly is available through NCBI GenBank under accession number ON260813.
